# Supplementary material for: Bridging Theories for Ecosystem Stability Through Structural Sensitivity Analysis of Ecological Models in Equilibrium
Source: Acta Biotheor. 2022 Jun 23;70(3):18. doi: 10.1007/s10441-022-09441-7 (PMC9225980; doi:10.1007/s10441-022-09441-7)
Supplement: Supplementary file 2 — Supplementary file2 (PDF 350 kb) [file 10441_2022_9441_MOESM2_ESM.pdf]

Bridging theories for ecosystem stability through structural sensitivity analysis of ecological models in equilibrium

Jan J. Kuiper, Bob W. Kooi, Garry D. Peterson & Wolf M. Mooij

Corresponding author: jan.kuiper@su.se

Acta Biotheoretica

Online Resource 2

Results of the Rosenzweig-MacArthur-Scheffer (RMS) model and the linearized version ($RMS_{(LV)}$) for increasing carrying capacity (*K*).

The pattern that the increasing productivity of the system ends up only in increasing biomass of the consumer has been lost (ESM2 Fig. 1a, line A*) and the model shows a more complex response of resource and consumer abundance to enrichment. In line with the LVV and RM model, the RMS model first goes through a transcritical bifurcation when increasing *K* (ESM2 Fig. 1a, K = 1.0), and shows a stable equilibrium thereafter. At a value of K = 3.39, however, a saddle-node bifurcation occurs and the equilibrium densities of resources and consumers suddenly switch to a different level, lower for the resource and higher for the zooplankton (ESM2 Fig. 1a). At decreasing carrying capacity, this switch takes place at a lower value of K = 3.12 and hence the system shows hysteresis and alternative stable states also for *K*. In addition to these alternative stable states, the system shows a supercritical Hopf bifurcation at K = 3.89 and the model shows stable limit cycles as its dynamic behavior at higher carrying capacities (ESM2 Fig. 1b).

For the linearized RMS model version $RMS_{(LV)}$, when increasing *K*, the model does show the transcritical bifurcation at the value of 1.0 but thereafter only shows stable focus for higher values of the carrying capacity (ESM2 Fig. 1b).

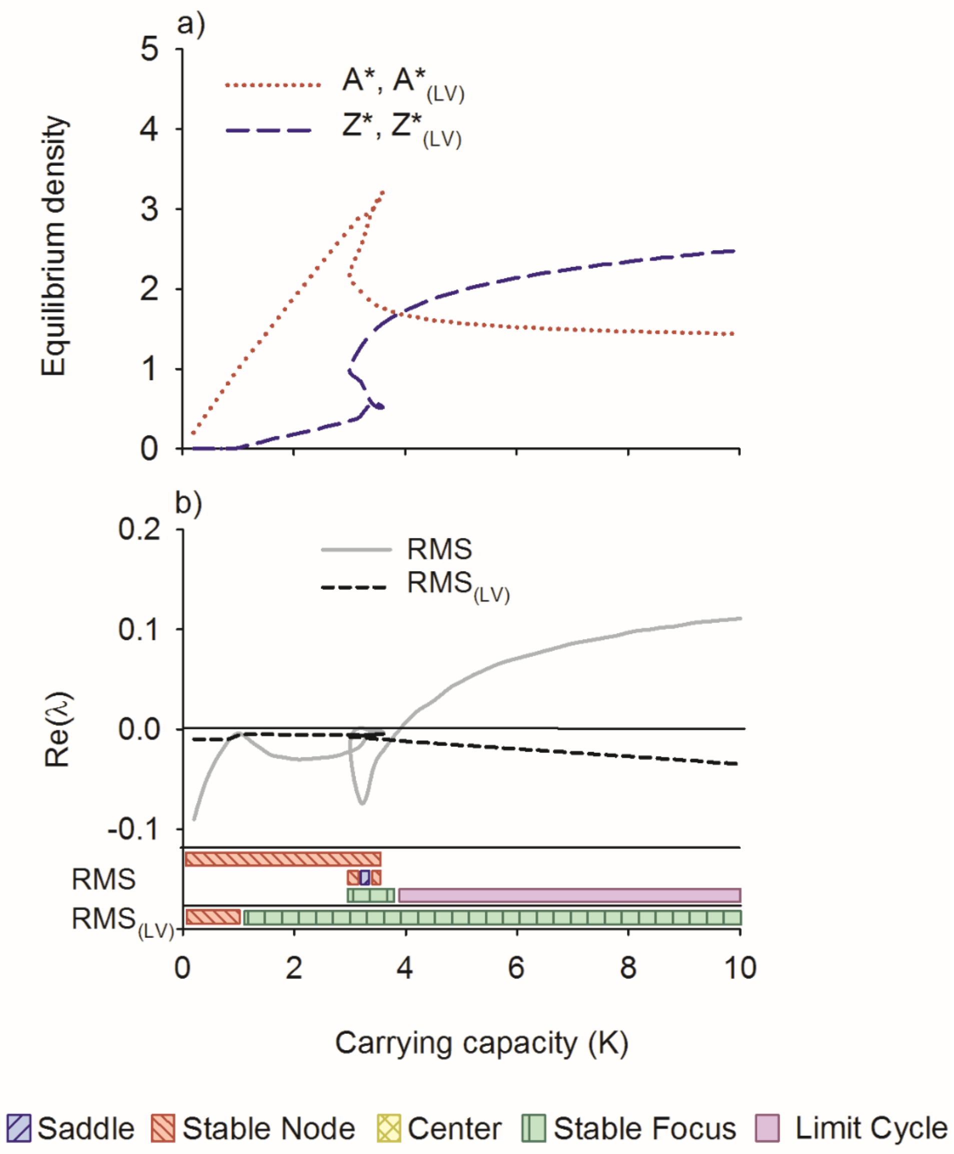

ESM2 Figure 1. Equilibrium densities (a) and the maximum real part of the eigenvalues (b) of the original Rosenzweig-MacArthur-Scheffer (RMS) and the linearized ($RMS_{(LV)}$) model along a gradient of increasing carrying capacity (K). Also the mode of behavior along the gradient is depicted. Parameters are $r = 0.5$, $g = 0.4$, $a = 0.6$, $l = 0.01$, $e = 0.6$, $m = 0.15$, $F = 0.05$ and $z = 0.5$.
