# Supplementary material for: Bridging Theories for Ecosystem Stability Through Structural Sensitivity Analysis of Ecological Models in Equilibrium
Source: Acta Biotheor. 2022 Jun 23;70(3):18. doi: 10.1007/s10441-022-09441-7 (PMC9225980; doi:10.1007/s10441-022-09441-7)
Supplement: Supplementary file 3 — Supplementary file3 (PDF 379 kb) [file 10441_2022_9441_MOESM3_ESM.pdf]

Bridging theories for ecosystem stability through structural sensitivity analysis of ecological models in equilibrium

Jan J. Kuiper, Bob W. Kooi, Garry D. Peterson & Wolf M. Mooij

Corresponding author: jan.kuiper@su.se

Acta Biotheoretica

Online Resource 3

Note on bifurcation analysis of RMS model with increasing maximum intake rate of the top consumer *F*

A global bifurcation (homoclinic connection) occurs between *G* lower *F* (*F* = 0.0784) and *G* higher *F* (*F = 0.2401*) (ESM3 Fig. 1). Between *T* (*F* = 0.0758) and *G* lower *F* (*F* = 0.0784) there is coexistence of a stable equilibrium and a stable limit cycle. For higher *F* there is coexistence of a stable equilibrium and a stable limit cycle between *G* (*F* = 0.2401) and *H* (*F* = 0.2404) and coexistence of two stable equilibria between *H* (*F* = 0.2404) and *T* (*F* = 0.2408).

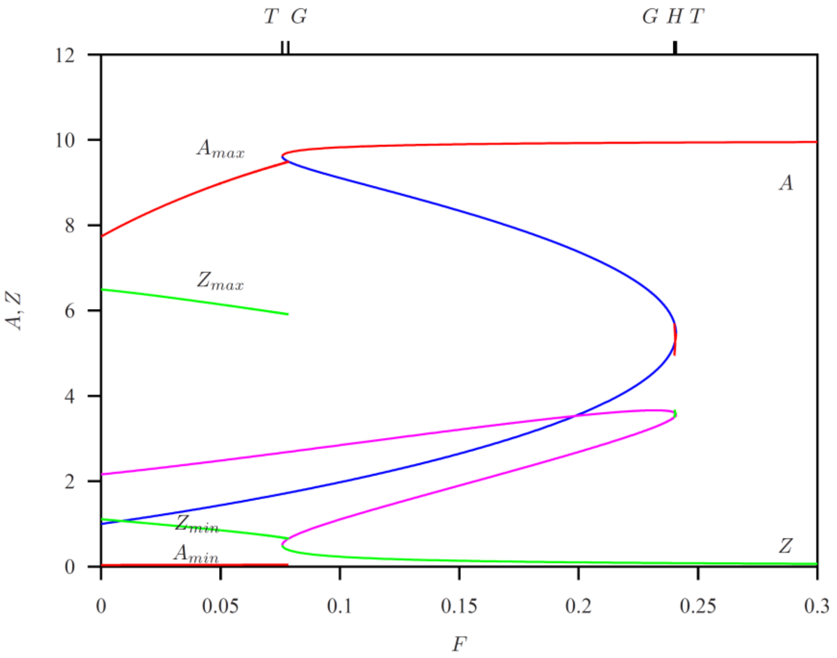

ESM3 Figure 1. Equilibrium densities *A* (red stable, blue unstable), *Z* (green stable, magenta unstable). In addition maximum *Amax*, *Zmax* values and minimum *Amin*, *Zmin* values of stable limit cycles for the RMS (Rosenzweig-MacArthur-Scheffer) model are shown. The carrying capacity *K* = 10.

The ESM3 Figure 2 is a detail of ESM3 Figure 1 where *F* ∈ [0.240 : 0.2408]. This shows that for a small interval below the top saddle-node there is a stable limit cycle. But this limit cycle disappears at the global homoclinic bifurcation *G*. In this interval there are two alternative states.

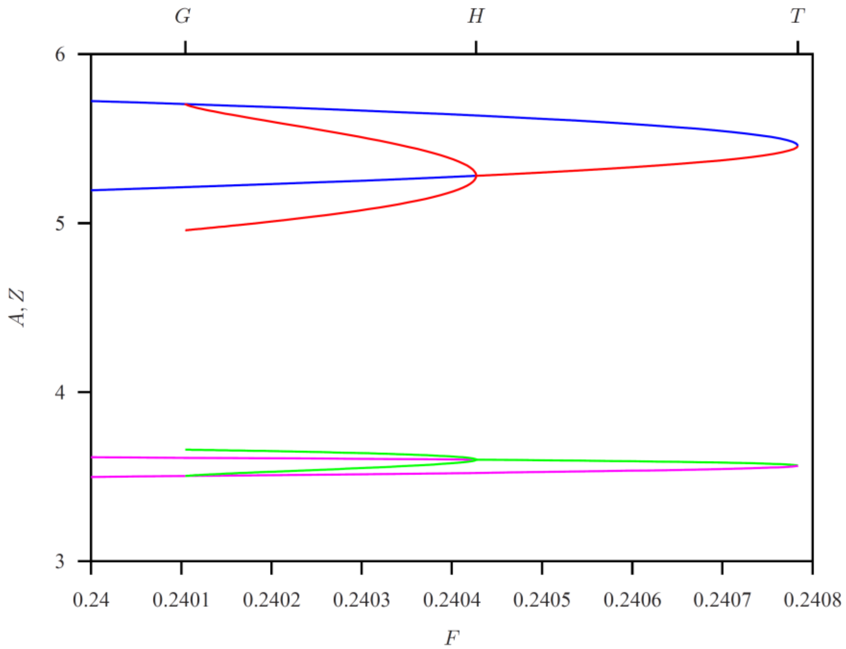

ESM3 Figure 2. Detail of ESM3 Figure 1 for $F \in [0.240 : 0.2408]$. Equilibrium densities *A*, *Z* and maximum *Amax*, *Zmax* values and minimum *Amin*, *Zmin* values of stable limit cycles for the RMS (Rosenzweig-MacArthur-Scheffer) model. The carrying capacity K = 10. Same color case as in ESM3 Figure 1.
